# Power boosts reliance on preferred processing styles

**DOI:** 10.1007/s11031-016-9548-8

**Published:** 2016-02-29

**Authors:** Małgorzata Kossowska, Ana Guinote, Paweł Strojny

**Affiliations:** Jagiellonian University, Kraków, Poland; University College London, London, UK; Pedagogical University of Cracow, Kraków, Poland

**Keywords:** Power, Systematic or heuristic processing, Memory for schema-consistent information, Stereotyping, Cognitive complexity

## Abstract

A significant amount of research has proposed that power leads to heuristic and category based information processing, however, the evidence is often contradictory. We propose the novel idea that power magnifies chronically accessible information processing styles which can contribute to either systematic or heuristic processing. We examine heuristic (vs. systematic) processing in association with the need for closure. The results of three studies and a meta-analysis supported these claims. Power increased heuristic information processing, manifested in the recognition of schema consistent information, in the use of stereotypical information to form impressions and decreased the complexity of categorical representations, but only for those participants who, by default, processed information according to simplified heuristics, i.e., are high in need for closure. For those who prefer this processing style less, i.e., low in need for closure, power led to the opposite effects. These findings suggest that power licenses individuals to rely on their dominant information processing strategies, and that power increases interpersonal variability.

## Introduction

Research has shown that power affects diverse psychological phenomena associated with increased reliance on heuristics (i.e., rules of thumb that can inform judgment, see Keltner et al. 2003), and category based thinking (Fiske 1993). Power holders are often socially inattentive, they rely on stereotypes (Fiske 1993; Guinote and Phillips 2010), sexualize others (Bargh et al. 1995), and fail to adopt another’s perspective (Chen et al. 2009; Galinsky et al. 2006). It has also been proposed that power licenses individuals to act at will, and gives them the freedom of self-expression (Kraus et al. 2011; Overbeck et al. 2006), such as the tendency to express enduring attitudes (Anderson and Berdahl 2002) and other chronically accessible constructs (Chen et al. 2001; Guinote et al. 2012). In the present article, we expand these findings to the domain of information processing strategies. We propose that power magnifies chronically accessible information processing styles, and that this in turn affects the extent to which people rely on heuristics. Thus, instead of arguing that power leads to a committed way of processing information (e.g., heuristic, systematic), we argue that power licenses individuals to use their default strategies.

In this paper we focus on the preference for heuristic (vs. systematic) processing, as these cognitive styles underlie many social cognitive phenomena (for an overview, see: Chaiken and Ledgerwood 2012). We examine heuristic (vs. systematic) processing in association with the need for closure, defined as the need to avoid ambiguity by having an answer on a given topic (Webster and Kruglanski 1994). High need for closure is manifested in category-based, nonsystematic and heuristic information processing style, preference in predictability and quick decision-making (Driscoll et al. 1991; Kruglanski and Webster 1996). By contrast, low need for closure leads to heuristic processing style less and it is usually manifested in vigilant behavior that is based on a more systematic and effortful search for relevant information, its evaluation, and its unbiased assimilation (for an overview see Roets et al. 2015). Thus we propose that power increased heuristic information processing, but only for those participants who, by default, processed information according to simplified heuristics, i.e., are high in need for closure. For those who preferred to process information less heuristically, i.e., low in need for closure, power led to the opposite effects. We expect also that the lack of power may lead people to less spontaneously apply their typical information-processing styles.

### Power and processing styles

It has been extensively argued that there is a link between power and increased heuristic processing (i.e., the use of simplified rules of thumb to form judgments, see Fiske 1993; Keltner et al. 2003). This proposition derives from the assumption that power holders are cognitive misers, unmotivated to deploy attention, especially in the social domain. Consistent with this notion, power holders have been found to use simplified, category-based information, such as stereotypes, to make judgments (Fiske,^1993^; Guinote and Phillips 2010). For example, upon reading information about social targets who belong to different ethnicities, individuals with power paid relatively more attention to information that was consistent with the national stereotypes of the targets compared to stereotype-inconsistent information. This was not the case for participants in a control or powerless position (Fiske 1993; Guinote and Phillips 2010). Similarly, compared to powerless individuals, when making social judgments, power holders relied more on their own vantage point (Chen et al. 2009; Galinsky et al. 2003), and on information that easily came to mind (e.g., ease-of-retrieval, Weick and Guinote 2008).

In spite of this evidence, a number of studies have shown that power holders do not always use schematic, effortless processes to guide their attention, judgments and actions. For example, Ebenbach and Keltner (1998) demonstrated that while participants with power tended to use heuristic, effort-saving strategies when making judgments about the attitudes of an ideological opponent, this was not the case when they experienced negative emotions associated with the ideological conflict. Negative emotions trigger systematic processing (see Schwarz and Clore 2003), and enhanced the accuracy of the judgments. Similarly, Overbeck and Park (2001) demonstrated that in interactions marked with a sense of responsibility, power enhanced attention and memory for the personal attributes of the interaction partners. Guinote et al. (2012) proposed a single mechanism to account for the contradictory response tendencies found in power holders. They argue that power increases reliance on accessible constructs and scripts (i.e., those that have a low threshold of activation) regardless of whether they are chronically accessible or temporarily activated by the states and goals of the power holder or by the environment.

Past research on the links between power and dispositions focused on trait-like chronically accessible constructs and scripts stored in memory. In the present article, we argue that not only trait related aspects of the self but also information processing styles are capable of being affected by power. Specifically, we argue that power licenses individuals to rely on their preferred ways of processing information (heuristic or systematic). Because people in powerful positions feel free to act at will and in authentic ways (Kraus et al. 2011), they do not have the need to constrain the use of their processing styles. In contrast, the lack of power may lead people to less spontaneously apply their typical information-processing styles. This notion is consistent with the finding that lack of power decreases self-expression. For example, individuals who lacked power felt obliged to smile, and smiled in less authentic ways compared to power holders (Hecht and LaFrance 1998). Similarly, studies focusing on eating behavior found that the eating behavior of power holders was guided by their feelings of hunger and how appetizing the food was, while for powerless individuals there was no relationship between eating and their feelings of hunger or the attractiveness of the food (Guinote 2010).

## Overview of the study

We expect that along with the freedom from constraints, the ability to act at will (Overbeck et al. 2006), and increased confidence (Petty et al. 2007), power holders may more freely rely on heuristic information processing, if they typically prefer this information processing style (i.e., if they are high in the need for closure). However, they should engage less on this information processing style if this is not their default mode (i.e., if they are low in the need for closure). Moreover, as lack of power usually leads people to less spontaneously rely on their dispositions, they also may be less prone to apply their typical information-processing style. Thus, in this condition we do not expect any relationship between need for closure and processing style.

To test these hypotheses we conducted three studies focusing on memory for schema-consistent information, stereotypical impression formation, and the construction of simple categories as core examples of heuristic processing (e.g., Fiske and Neuberg 1990; Fiske and Taylor 1984; Kruglanski and Mayseless 1988; Schroder et al. 1967; Van Hiel and Mervielde 2003). We expect that powerful people who typically process information according to simple heuristics (high need for closure) will recognize more schema-related information, use more stereotypical information to form impressions about a target group, and create less complex social categories, compared to those who prefer to process information in less heuristic and more systematic way (low need for closure). If our hypotheses that power magnifies default processing is true, we should also observe more less heuristic thus more systematic processing under power among low need for closure participants. Crucially, the influence of default processes on social judgments should be more pronounced for power holders than for individuals who do not have power.

## Study 1

In Study 1 we tested the hypothesis that power amplifies the links between chronic processing strategies and preferences for schema-consistent information. A preference for heuristic processing manifests in increased attention and memory for schema-consistent compared to schema-inconsistent information (see Fiske and Neuberg 1990). Importantly, we expected this effect to be especially pronounced among high (vs. low) power participants.

Positive mood boosts default information processing strategies (Hunsinger et al. 2012), and power has been associated with positive mood (Keltner et al. 2003). Thus, to check the possibility that the effects of power derive from differences in mood, mood was assessed in this study.

### Method

#### Participants

A total of 50 students (36 females and 14 males; *M*_age_ = 16.6, *SD* 0.84) participated in the study on a voluntary basis.^1^ Two participants failed to complete the measures, thus their results were excluded from the analyses. Participants were randomly assigned to the powerful or the powerless conditions.

#### Materials and procedure

Participants took part in the experiment in small groups. At the beginning of the session, participants completed the Need for Closure Scale (Webster and Kruglanski 1994) to assess their preferred information processing styles (heuristic vs. systematic). One of the subscale, *Decisiveness*, has been considered as an unreliable measure of motivation, and was replaced with six items developed by Roets and van Hiel (2007). Answers were given on six-point scales, from (1) completely disagree, to (6) completely agree. From these measures, a single scale was formed (Cronbach’s *α* = .81, *M* = 3.56, *SD* 0.41). Higher mean values indicate a higher preference for heuristic processing.

Subsequently, participants were informed that they would work on two independent studies. The first study allegedly investigated the perception of past events. The second focused on the ways people form impressions of the personalities of other people. First, power was manipulated by asking participants to report either a past event in which they had power over someone, or a past event in which someone had power over them (Galinsky et al. 2003). The written report was followed by a manipulation check that read ‘‘Now we would like to know how much in charge you were in this situation.” Answers were given on a 6-point scale ranging from 1 (not at all) to 6 (very much). Participants also reported their mood on a single 6-point scale, from (1) very bad, to (6) very good.

The experimenter, who was unaware of hypotheses or conditions, then introduced the second, ostensibly unrelated, study. To measure preferences for schema-consistent information we used a classic task (Neuberg and Fiske 1987; Sentis and Burnstein 1979) that asks participants to form impressions about target people. Participants were given the written instruction, that informed that they would be presented with information about two different persons, whose friendliness had been assessed in a previous study. To help participants form a hypothesis about the two targets, one was described as “very friendly” by more than 80 % of the previous participants, and the other was described as “very unfriendly” by more than 80 % of the previous participants. Participants were then informed that they would be presented with a few statements describing each target. Each item was presented on a separate display (e.g. “Tom (friendly): Volunteered to care for lonely old people.”). Participants were tasked with assessing the extent to which each piece of information confirmed the trait *friendly* of a given person on a scale from 1 (“Does not confirm”) to 6 (“Fully confirms”). Participants were presented with 30 sentences (15 per target). Both sets comprised five items consistent with the trait, five items inconsistent with the trait, and five items irrelevant to the trait. An example of a friendly-consistent item was: “Volunteered to care for lonely old people.” An example of an inconsistent item was: “Refused to talk with fellow passengers on an organized trip.” An irrelevant item is illustrated by the sentence: “Works as an accountant.” Sentences were presented in random order. For the “unfriendly person,” the information presented was analogous. Afterwards participants were presented with a surprise recognition task for the information they had read about the target people. The task presented participants with 45 statements in random order, of which 15 were “friendly” and 15 “unfriendly”. The fifteen other statements were new. Among them 5 items were consistent, 5 inconsistent and 5 irrelevant. Participants were asked to assess the extent to which each sentence describes the target person on 6 points scale, from (1) “the sentence certainly did not described target person,” to (6) “the sentence certainly described target person.” The number of points assigned to schema-consistent versus to schema-inconsistent and irrelevant sentences correctly recognized was calculated and used as an indicator of heuristic information processing. Participants were then thanked, debriefed and dismissed.

### Results and discussion

Participants indicated how much they thought they were in charge of the situation they reported. An independent *t* test revealed that participants in the powerful condition felt more in charge of the situation they recalled (*M* = 4.75; *SD* 0.79) than participants in the powerless condition (*M* = 2.88; *SD* 1.27), *t*(48) = 6.15, *p* < .001, 95 % CI [−2.47, −1.25]. The need for closure was equally distributed among conditions (*t*(48) = 0.99, *p* = .26).

**Fig. 1:**
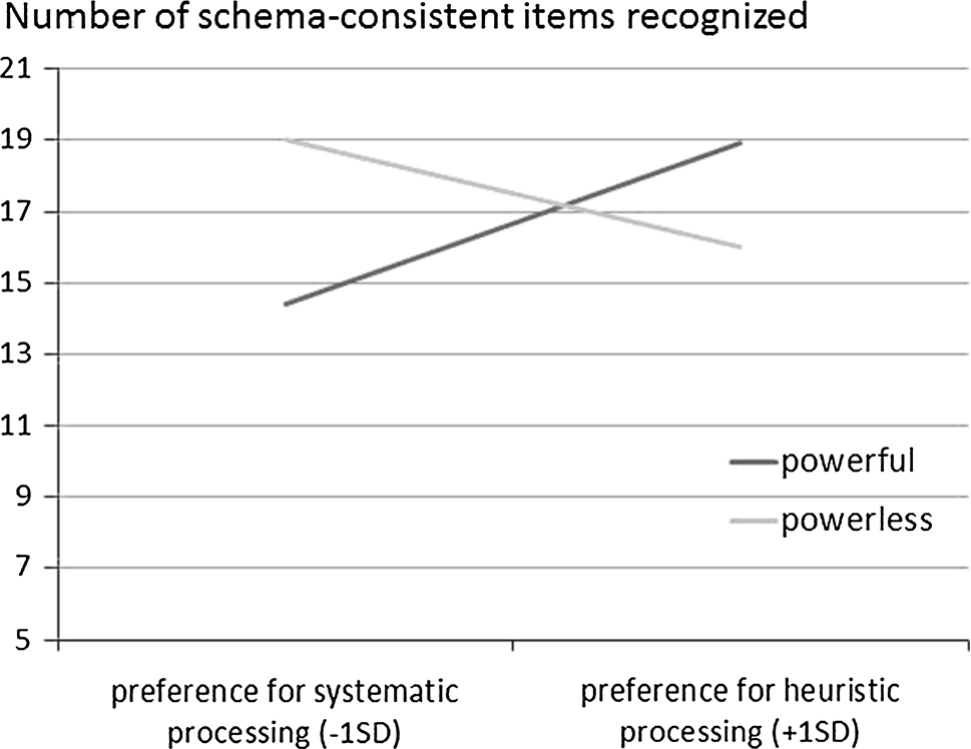
Regression lines showing memory for schema-consistent items as a function of processing style and power

No gender or age differences were found, therefore, these variables were not considered in further analyses. We found significant main effect of power (*b* = −2.15; *t*(48) = 2.12; *p* = .04). Main effect of need for closure was non-significant (*b* = .06; *t*(48) = 0.95; *p* = .35). To examine the effects of power and processing style on schema-consistent memory, we run regression analysis with power as predictor and need for closure as moderator, using the PROCESS program (Hayes 2013, model 1). The experimental conditions were coded with −1 (powerless) and 1 (power). We calculated the effect of power on the schema-consistent memory for low and high values (−1 *SD*, +1 *SD*) of the moderator. The interaction between preference for heuristic processing, i.e., need for closure, and power on schema-consistent recognition was significant (*R*^2^ = .15; *b* = .12; *p* = .017, 95 % CI [0.02, 0.22]). The interaction pattern is depicted in Fig. 1.

The analyses indicated that preference for heuristic processing, operationalized as high need for closure, was positively related to memory for schema-consistent information for powerful participants (*b* = .15, *p* = .03, 95 % CI [0.01, 0.28]) and non-significantly related to it among powerless participants (*b* = −.09, *p* = .20, 95 % CI [−0.24, 0.05]). Moreover, participants low in the need for closure recognized significantly more schema-consistent items in the powerless condition, as compared to the powerful condition (*t*(48) = 2.88; *p* < .01); while high need for closure participants recognized more of these items in the powerful, as compared to powerless, condition (*t*(48) = 2.11; *p* = .04). The mood ratings did not differ between high-power and low-power participants, *t*(48) = 1.02, *p* = .39, 95 % CI [−0.94, 0.30].

To check the possibility that the valence of the induced schema (“friendly” vs. “unfriendly”) influences the obtained effects, we performed a three-way interaction between valence, experimental condition (powerless vs. power) and need for closure. The main effects of valence (*b* = −1.08, *p* = .40, 95 % CI [−3.64; 1.46] as well as the interaction (*b* = −.07, *p* = .37, 95 % CI [−2.36; 0.98]) were not significant.

The results of Study 1 supported our predictions. Compared to lacking power, having power enhanced the use of default information processing styles. Powerful participants who preferred heuristic processing (those high in the need for closure) recalled more schema-consistent information, but those who preferred this processing style less (those low in the need for closure) recalled less of this type of information. In the low power condition, chronic processing strategies did not influence memory. We did not find the effect of power on mood, therefore, mood could not explain the effects of power. Numerous researchers have argued that power is expected to bias attention toward positive information (i.e., rewards) and the powerless toward negative information (i.e., potential punishments; see Anderson and Berdahl 2002; Galinsky et al. 2014; Gruenfeld et al. 1998; Keltner et al. 2003; Kunstman and Maner 2011). However, we didn`t find any differences in overall heuristically-consistent recall for the target when labeled as friendly versus unfriendly. This finding provides evidence that the effects are not driven by heightened attention to positive or negative information.

## Study 2

Although the results of Study 1 provided support for the hypothesis that power magnifies the use of default processing styles, the study did not include a control condition, and it was not clear that power was driving the effects. To verify that the effects obtained in Study 1 derive from having power, Study 2 included a control condition. In this study, we used stereotyping as a manifestation of heuristic information processing style (e.g., Chen and Chaiken 1999). The study tested the hypothesis that preferred processing styles, operationalized as need for closure, will guide the degree to which a target group is perceived in a stereotypic way for participants in the powerful, compared to control, condition.

Understanding the relationship between power and stereotyping is important given the contradictory findings in past research (e.g., Fiske 1993; Overbeck and Park 2001; Weick and Guinote 2008). We expected that, in the powerful (vs. control) condition, those participants who prefer heuristic processing, as indexed by the high need for closure, would rely more on the stereotypes of the target group. Participants who do not prefer heuristic processing (low in need for closure) would rely on the stereotypes less. The possible role of mood in this process also was examined.

### Method

#### Participants

A total of 52 students (35 females and 17 males; *M*_age_ = 19.86, *SD* 1.41) participated in the study on a voluntary basis. Participants were randomly assigned to the powerful or control conditions. Ten participants did not complete the dependent measure, as they received questionnaires with missing pages, thus their data were excluded from the analyses.

#### Materials and procedure

To identify participants’ default heuristic information processing styles they completed five subscales of the Need for Closure Scale (Webster and Kruglanski 1994). The decisiveness subscale was not considered because it has been recognised to measure the ability to impose closure rather than the motivation for closure (Roets and Van Hiel 2007). A higher mean score (Cronbach’s *α* = .77, *M* = 3.82, *SD* 0.64) indicates a higher preference for heuristic processing. Similarly to Study 1, power was manipulated by asking participants to report a past event in which they had power over someone. Participants in the control condition were asked to report what they did the day before. Subsequently, participants completed the same manipulation check as in Study 1, and they reported their mood using 6-point scales, from (1) very bad to (6) very good.

The experimenter, who was unaware of hypotheses or conditions, then introduced an ostensibly unrelated study on person perception. Participants were given a list of 13 attributes related to the stereotype of Gypsies, as tested in a previous study by Kofta and Narkiewicz-Jodko (2003). The attributes were: unreliable, educated, lazy, friendly, competent, moral, dishonest, family man, orderly, neat, intrusive, insolent, filthy. Participants were asked to assess on a 7 point scale (1—completely do not agree, 7—completely agree) to what extent they agreed that typical Gypsies had these characteristics. Positive attributes were reverse coded. Averaged assessments of the attributes served as an index of negative stereotypes (Cronbach’s α = .71; *M* = 3.46; *SD* 0.60). Participants were subsequently thanked, debriefed and dismissed.

### Results and discussion

Participants indicated how much they thought they were in charge of the situation they reported. To investigate whether the manipulation of power was successful, an independent *t* test (power vs. control) was conducted on this measure. As expected, participants in the powerful condition felt more in charge of the situation they recalled (*M* = 4.74; *SD* 1.25) than participants in the control condition (*M* = 3.78; *SD* 1.62), *t*(41) = 2.21; *p* = .03, 95 % CI [−1.82, −0.08]. The need for closure was equally distributed among conditions (*t*(41) = 0.04, *p* = .69).

**Fig. 2:**
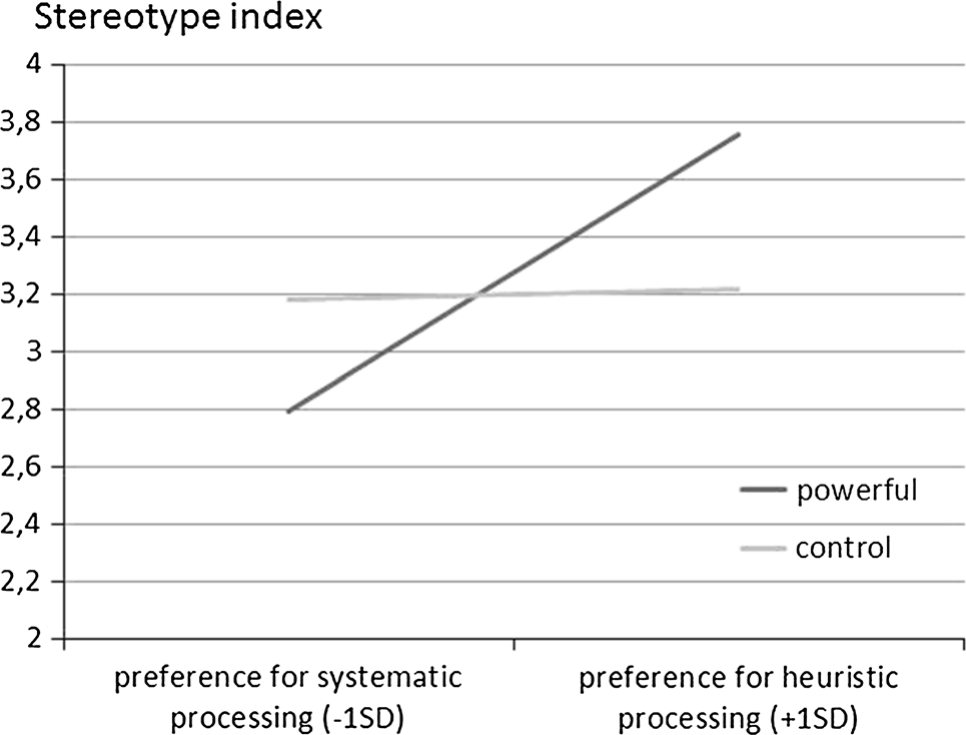
Regression lines showing the stereotype index as a function of processing styles and power

No gender or age differences were found, therefore, these variables were not considered in further analyses. The main effect of power was non-significant (*b* = −.04; *t*(41) = 0.17; *p* = .68).The main effect of the need for closure was significant (*b* = .34; *t*(41) = 2.14; *p* = .039). To examine the joint effects of power and default processing styles, we used the PROCESS program (Hayes 2013, model 1). As in Study 1, we run regression analysis with power as predictor and need for closure as moderator. The experimental conditions were coded with −1 (control) and 1 (power). We calculated the effect of power on the DV for low and high values (−1 *SD*, +1 *SD*) of the moderator. Crucially, there was a significant interaction between heuristic processing styles and power (*R*^2^ = .28; *b* = .44, *p* = .01, 95 % CI [0.10, 0.77]). The interaction can be seen in Fig. 2.

Simple slope analyses indicated that the preference for heuristic processing (high need for closure) was positively related to the stereotype index for powerful participants (*b* = .92, *p* < .001, 95 % CI [0.41, 1.43]) and non-significantly related to it among participants in the control condition (*b* = .04, *p* = .86, 95 % CI [−0.39, 0.47]). Moreover, participants low in the need for closure did not differ in stereotyping in both conditions (*t*(41) = 1.85; *p* = .07); while participants high in the need for closure stereotyped more in the powerful, compared to the control condition (*t*(41) = 2.27; *p* < .01). Power did not affect mood, *t*(41) = 0.42, *p* = .67. There is also no significant correlation between mood and stereotyping (*r* = .06*; p* = .71).

The results of Study 2 demonstrated once more that power increases the use of preferred processing styles. Power may lead to more or less stereotyping depending on the individuals’ cognitive preferences, i.e., need for closure. Powerful participants, who preferred more heuristic strategies (i.e., those high in the need for closure), relied more on stereotypes compared to those who preferred heuristic processing less (i.e., those low in the need for closure). Again, we did not find the effect of power on mood, therefore, mood could not explain these effects.

## Study 3

Study 3 further tested the links between power and processing styles in the domain of cognitive complexity. In the present context, cognitive complexity refers to the capacity to construe social behavior in multidimensional ways, a capacity that requires less heuristic and more systematic processing (Schroder et al. 1967). We hypothesized that, for participants in the powerful condition, the higher their need for closure, the less complex will be the categories they construe to describe social targets. This should not be the case for participants in the powerless condition.

### Method

#### Participants

A total of 77 students (34 females and 43 males; *M*_age_ = 22.12, *SD* 2.12) participated in the study on voluntary basis. Participants were randomly assigned to the powerful and powerless conditions.

#### Materials and procedure

As in the previous studies, four subscales of the Need for Closure Scale (Webster and Kruglanski 1994) were used to identify participants’ default processing strategies. Due to the low reliability (Cronbach’s α = .25), the Closed-mindedness subscale^2^ was excluded from the analyses, and the overall index was calculated using only three subscales (Cronbach’s *α* = .76, *M* = 4.43, *SD* 0.73). A higher mean score indicated a higher preference for heuristic processing. Power was manipulated as it was in Study 1. Upon completion of the power manipulation, participants filled in the manipulation check and reported their mood.

The experimenter, who was unaware of hypotheses or conditions, then introduced a second, ostensibly unrelated, study. Cognitive complexity was measured using an object sorting task (Scott 1962), in which participants have to place objects into meaningful categories. Participants were asked to arrange a list of 28 nations into categories, which they thought belonged together, and to indicate what they thought the nations had in common. For example, from a list of nations, Japan and England might be grouped together as island nations. This procedure was continued until the number of categories of each subject was exhausted. Cognitive complexity is measured by the number of distinctions made in the category system. The greater the number of different attributes ascribed to the objects, the higher the complexity score. The cognitive complexity score was calculated with a formula suggested by Scott (1962) and based on information theory.^3^ Participants were subsequently thanked, debriefed and dismissed.

### Results and discussion

An independent *t* test (power vs. powerless) indicated that participants in the powerful condition felt more in charge of the situation they recalled (*M* = 4.20; *SD* 1.30) than participants in the powerless condition (*M* = 2.43; *SD* 1.28), *t*(74) = 5.89; *p* < .001, 95 % CI [1.18, 2.36]. Thus, the manipulation effectively induced power differences. The need for closure was equally distributed among conditions (*t*(74) < 0.25, *p* = .80).

**Fig. 3:**
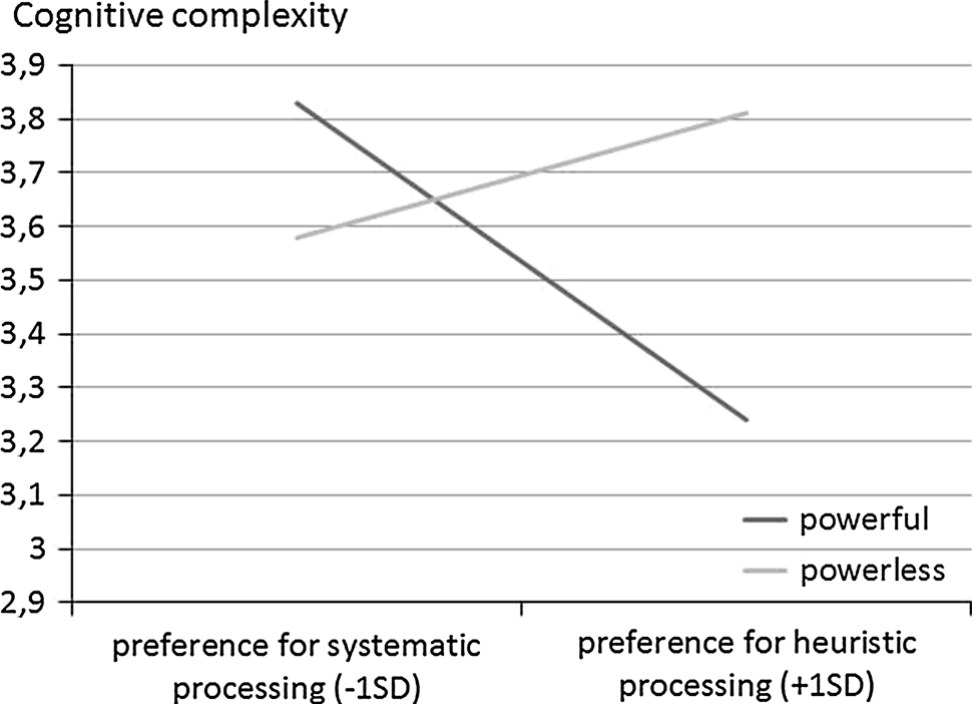
Regression lines showing cognitive complexity as a function of processing styles and power

No gender or age differences were found, therefore, these variables were not considered in further analyses. Main effects of power (*b* = −.10; *t*(74) = 1.08; *p* = .28) and need for closure (*b* = −.11; *t*(74) = .85; *p* = .40) were non-significant. To examine the effects of power and processing styles on the dependent variable, as in previous studies we run regression analysis with power as predictor and need for closure as moderator, using the PROCESS program (Hayes 2013, model 1). The experimental conditions were coded (−1 powerless/1 power). We calculated the effect of power on the DV for low and high values (−1 *SD*, +1 *SD*) of the moderator. The results of the analysis revealed a significant interaction between preferred processing styles (i.e., high vs. low need for closure) and power on the cognitive complexity index (*R*^2^ = .08, *b* = .26, *p* = .03, 95 % CI [0.02, 0.50]). The interaction is illustrated in Fig. 3.

Because we were interested in the relationship between preferred processing styles and cognitive complexity in the powerful and powerless conditions separately, we performed simple slope analyses. The analyses indicated that the preference for heuristic processing, i.e. need for closure, was negatively related to cognitive complexity for people in the powerful condition (*b* = −.36, *p* = .02, 95 % CI [−0.56, −0.06]) and unrelated to it for participants in the powerless condition (*b* = .16, *p* = .38, 95 % CI [−0.20, 0.54]). Moreover, low need for closure did not differentially affect the complexity of the categories construed by participants in powerful and powerless condition (*t*(74) = 1.45; *p* = .15). In contrast, participants high in the need for closure used less complex categories in the powerful condition, compared to the powerless condition (*t*(74) = 2,43; *p* = .02). Power did not affect mood, *t*(74) < 0.30; *p* = .76.

The results of Study 3 supported the hypothesis. In the powerful condition, those participants with a preference for more heuristic processing expressed less complex social structures, compared to those participants with a preference for less heuristic processing. In the powerless condition, the pattern of results was non-significant. Thus, we conclude that power magnifies reliance on idiosyncratic processing styles. Conversely, the lack of power may lead individuals to refrain from using default processes. Again, we did not find the effect of power on mood. Mood can not therefore explain the abovementioned effects.

## Study 1–3: Meta-analysis

Given that each study only differed in terms of the materials that were used, and that did other manipulations were not included, we report the integrated results using a meta-analysis of the three experiments (Cumming 2014). The meta-analysis was conducted using Comprehensive Meta-Analysis Software, on standardized regression coefficients and its standard errors. The analysis was performed on values of regression coefficients for the predictor (need for closure), obtained from simple slope analysis of interaction terms. So, in each study there were two separate predictor coefficients (one for each experimental condition). In each study we used different manifestations of heuristic processing as dependent measures (total *N* = 165). Across the three studies we have high power conditions, across two studies low power conditions and in one a control condition. As we were mainly interested in the relationship between the preference for heuristic processing (measured via the need for closure) and its manifestation in the powerful and powerless/control conditions separately, we integrated the results for the high power conditions from three studies and for low power conditions from two studies. We did not include the results from the control condition to make the results more clear. Thus, we analyzed data from three studies, in two within-study subgroups (for the high power condition we included effects from three studies; for low power condition we included effects from two studies). We used the random-effects model, as it is appropriate and more realistic in this case (Schmidt et al. 2009). It assumes that the population means estimated by the different studies are randomly chosen from a superpopulation with standard deviation of τ (Cumming 2014).

**Fig. 4:**
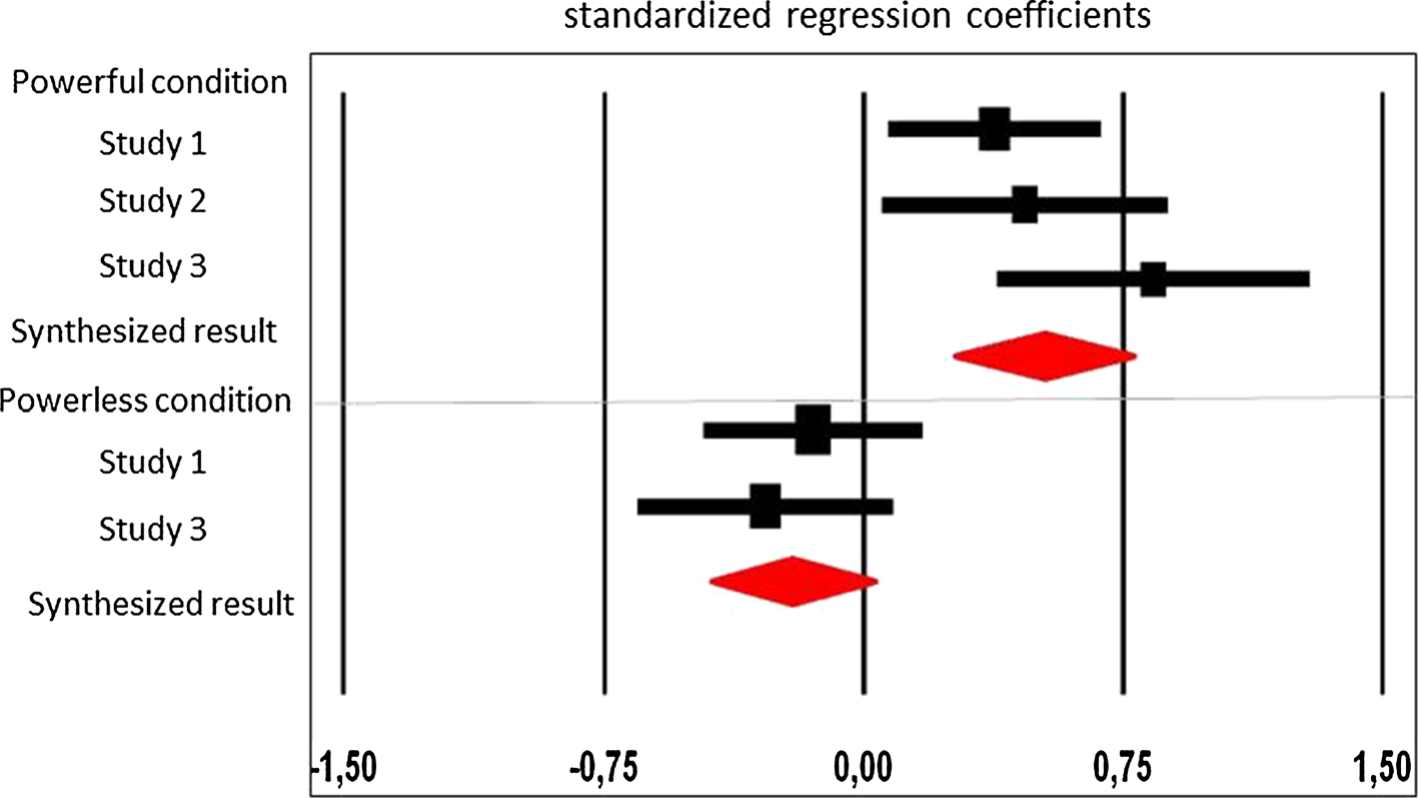
Meta-analysis of three current studies. *Error bars* represent 95 % confidence intervals

The calculated effect size and confidence interval of the heuristic processing manifestation is reported in Fig. 4. The heterogeneity of effects sizes was not statistically significant (high power: *Q*(2) = 3.72, *p* = .16, *I*^2^ = 46.17 %; low power: *Q*(1) = 0.28, *p* = .60, *I*^2^ = 0.00 %). As predicted, the analysis indicated that preference for heuristic processing was positively and significantly related to the heuristic processing expression for participants from the powerful condition (*β* = .53, *SE* .13, *p* < .001, 95 % CI [0.22, 0.83]) and negatively but not statistically significantly related to it among participants from the powerless condition (*β* = −.21, *SE* .10, *p* < .01, 95 % CI [−0.455, 0.028]). However, the difference between these two conditions was highly significant, as indicated by high the between-group variance component *Q*(1) = 13.76, *p* < .001.

## General discussion

In three studies we found that across a variety of domains, such as memory for schema-consistent information, stereotyping, and cognitive complexity, situationally induced power consistently increased reliance on default information processing styles. Power increased the recognition of schema consistent information, the use of stereotypical information to form impressions, and decreased the complexity of categorical representations, but only for those participants who preferred to process information in a heuristic way prior to attaining power. For those who preferred to process information less heuristically and more systematically, power led to the opposite effects. These effects were not present for the control and powerless conditions. Together, these findings indicate that power accentuates the ways individuals typically process information.

A great deal of past research focused on the effects of power on information processing, and in particular, whether power increases the reliance on stereotypes (for reviews see Fiske and Berdahl 2007; Guinote 2013). Even though evidence suggests that this is often the case, the notion that power holders are cognitive misers, unmotivated to be socially aware should not be generalized. For example, it has been shown that power holders effectively pursue goals (Guinote 2007), and can pay close attention to subordinates when individuating information is relevant to the attainment of their goals (Overbeck and Park 2001). Guinote et al. (2012) explained the variability of power related findings, arguing that power leads to flexibility and situated responses, in line with accessible constructs, including those that are temporarily or chronically accessible (associated with dispositions). Expanding this notion to the present context, the findings reported here show that, similar to accessible declarative memory, accessible procedural memory regarding processing styles is also amplified by power. That is, instead of leading to a particular way of processing information, power seems to magnify the default, idiosyncratic processing strategies that individuals typically prefer. Therefore, consistent with past research (Guinote et al. 2002), power increased interpersonal variability. Part of the inconsistencies found in past research could derive from differences in the preferred processing styles of power holders, triggered by chronic response tendencies.

The present work focused on heuristic processing, assessed through the need for closure (Kruglanski et al. 2009). One limitation of the present research is that it did not include other information processing dimensions. For example, systematic processing will be better measured via need for cognition than low level of need for closure. We would expect power to magnify reliance on other default processing preferences, such as local or global, abstract or concrete, fast or slow (Kozhevnikov et al. 2014). Power holders’ sense of confidence and reliance on accessibility should facilitate the use of any default procedural strategies. These hypotheses await future research.

Future research also needs to consider how power and dispositions interact with environmental inputs, such as organizational goals, and with temporary states of the perceiver, such as emotions. Given the greater cognitive flexibility of power holders (Guinote 2007), we would expect them to be able to adapt processing strategies to salient goals or inner states. Research that simulated organizational contexts supports these claims, showing that power holders can be socially attentive or inattentive depending on whether the organization was person-centered or product-centered (Overbeck and Park 2006). Similarly, emotions shape the attentional strategies of power holders (Ebenback and Keltner 1998). Dispositions and context need to be considered in order to more fully understand the implications of these findings, namely that power enhances preferred information processing strategies.

## Footnotes

1 For each study, we aimed to collect at least 45 participants, based on a priori power analysis with medium effect size (f = 0.25) and power at .80. We did not conduct any statistical analyses before we finished collecting the data.

2 The effects found in Studies 1 and 2 persisted even when omitting the closed-mindedness subscale as in Study 3.

3 Complexity = log_2_n − 1/nΣ(n_i_log_2_n_i_), where n is the total number of countries, n_i_ is the number that appears in a particular combination of groups.
